# Transcranial Magnetic Stimulation Improves Executive Functioning through Modulation of Social Cognitive Networks in Patients with Mild Cognitive Impairment: Preliminary Results

**DOI:** 10.3390/diagnostics13030415

**Published:** 2023-01-23

**Authors:** Leonardo Sacco, Martino Ceroni, Deborah Pacifico, Giorgia Zerboni, Stefania Rossi, Salvatore Galati, Serena Caverzasio, Alain Kaelin-Lang, Gianna C. Riccitelli

**Affiliations:** 1 Neuropsychology and Behavioral Neurology Research Unit, Neurocenter of Southern Switzerland, EOC, 6900 Lugano, Switzerland; 2 Faculty of Biomedical Sciences, Università della Svizzera Italiana, 6900 Lugano, Switzerland; 3 Movement Disorders Unit, Neurocenter of Southern Switzerland, EOC, 6900 Lugano, Switzerland; 4 Department of Neurology, Inselspital, Bern University Hospital, 3010 Bern, Switzerland

**Keywords:** mild cognitive impairment, treatment, transcranial magnetic stimulation, cognition, executive functions

## Abstract

(1) Background: Patients with mild cognitive impairment (MCI) often present impairment in executive functions (EFs). This study aimed to investigate the effect of high-frequency repetitive transcranial magnetic stimulation (rTMS) on EFs in patients with MCI. (2) Methods: A prospective trial was conducted on 11 patients with MCI. Participants underwent 25 min of 20 Hz rTMS for ten days on the right temporo-parietal junction (RTPJ) and medial prefrontal cortex (MPFC). Before (T0) and after rTMS treatment (T1), global cognitive profile and EFs were investigated using the Montreal cognitive assessment (MoCA), trial making test (TMT) A and B, and frontal assessment battery (FAB). Depression symptoms were assessed using the geriatric depression scale (GDS). Statistical analysis included Wilcoxon signed-rank test. (3) Results: After treatment, patients showed a significant improvement in the MoCA EFs subtask (T0 vs. T1, *p* = 0.015) and TMT-B (T0 vs. T1, *p* = 0.028). Five MCI patients with EF impairment showed full recovery of these deficits. No significant changes in the GDS were observed. (4) Conclusions: rTMS stimulation over the TPJ and MPFC induced significant short-term improvements in EFs in MCI patients. These findings suggest that the TPJ and MPFC may be involved in the attention-executive skills to redirect attention toward behaviorally relevant stimuli.

## 1. Introduction

The term mild cognitive impairment (MCI) was introduced by Petersen et al. in 1997 to define a neuropsychological and clinical condition characterized by the appearance of cognitive disturbances reported by the patient or a reliable informant in the absence of significant repercussions on daily life and without overt dementia [1]. Accordingly, MCI formally designates an intermediate state, or a continuum, between normal aging and the diagnosis of dementia [2].

There are two types of MCIs, amnesic and nonamnesic; nonamnesic MCI may present deficits in single or multiple cognitive domains, such as memory, language, visuospatial abilities, processing speed, or executive functions (EFs); while in the amnesic form, the memory domain only becomes compromised [3].

MCI is, therefore, a clinical condition formally linked to age; its prevalence has been estimated at 6.7% for those 60–64 years, 8.4% for those 65–69 years, 10.1% for those 70–74 years, 14.8% for those 75–79 years, and 25.2% for those 80–84 years [4]. Moreover, Roberts and collaborators [5] highlighted that the incidence rate is higher for amnesic MCI (37.7/1000 person-years) than for nonamnesic MCI (14.7/1000 person-years), and the risk is more pronounced for men than women and individuals with ≤12 years of education.

For some authors [6], amnesic MCI precedes the development of Alzheimer’s disease (AD), whereas nonamnesic MCI probably anticipates other neurodegenerative processes such as frontotemporal dementia (FTD), dementia with Lewy bodies (DLB), or vascular dementia (VD). Although Devanand et al. [7] reported an annual progression rate of approximately 5% for dementia, other studies have highlighted that some MCI patients do not progress at all toward dementia [8] and even revert to normal [9], with an estimated reversion rate of 12.3% per year [10].

Although the progression from MCI to dementia is not clear, evidence suggests that it may depend on many concomitant factors such as mood-related depressive symptoms [11], anxiety [12], age [13], cardiovascular risk factors [14], typology, and severity of cognitive dysfunction. Accordingly, Brandt et al. [15] suggested that disorders of EFs in MCI patients represent a specific risk factor for developing dementia, and that patients affected by multidomain MCIs have different deficits of EF components with respect to the “pure” amnesic MCI.

Moreover, Van Dam et al. [16] also demonstrated attentional disorders in amnesic MCI. However, since EF disorders were more marked in multidomain MCI subjects, they should have been more at risk for developing dementia.

Scientific and clinical interest in the application of therapeutic interventions for MCI has increased. Since patients with MCI are at a high risk of developing dementia, they represent an ideal clinical target group to test and develop new therapeutic approaches in the early phase of disease progression [17]. Accordingly, it has been demonstrated that targeted clinical therapies can stop or at least slow down neurodegenerative progression, thus allowing the clinical state to be preserved as long as possible [18].

Unfortunately, there is still no high-quality evidence supporting an effective pharmacological therapy for MCI [19], except for Aducanumab, an amyloid beta-directed monoclonal antibody approved for the early stage of Alzheimer’s disease (AD) in the USA; however, further study of its efficacy is necessary [20]. For this reason, Petersen and colleagues [4] suggested that physical exercise and cognitive training may be more efficacious to improve the global functioning of MCI individuals. These results were confirmed by Chen and collaborators [21], who reported a slight benefit on EFs subsequent to exercise training.

Contrastingly, noninvasive brain stimulation (NIBS), such as transcranial direct current stimulation (tDCS) or repetitive transcranial magnetic stimulation (rTMS), has become a very promising approach in the treatment of different psychiatric and neurological disorders [22,23,24]. Birba et al. [25] described several NIBS approaches in patients with MCI and AD. However, the effectiveness of NIBS in improving cognitive functioning in this population is still far from being fully demonstrated. Furthermore, its mechanism of action is not clear; it may be related to the modulation of cortical plasticity, changes in brain blood flow, enzymatic activity, interactions between cortical and subcortical structures, and/or gene expression [26,27].

Indeed, TMS is more effective than tDCS in enhancing global cognition in MCI patients [28]. Moreover, TMS is safe and well-tolerated in MCI patients [29]. Furthermore, because the effects of rTMS are not restricted to the stimulation site, depending on the stimulation parameters, clinicians can induce effective modulations of remote and interconnected networks [30,31].

Based on this evidence, we applied an excitatory rTMS stimulation protocol involving two different regions: the temporo-parietal junction (RTPJ) and the medial prefrontal cortex (MPFC). Both areas are interconnected with the default mode network (DMN) and ventral attention network (VAN).

The DMN is usually known for the resting state [32], but it is also activated/modulated in high-level social/EF activities [33], while the VAN is particularly involved in reorienting attention to behaviorally relevant stimuli [34].

Accordingly, the idea is to first stimulate the RTPJ to activate part of the DMN and VAN to improve attentional control, as previous studies showed [35,36,37,38], and then to target the MPFC to focus on executive functioning in a state of alertness of the attentional networks. This site-related sequence might allow effective stimulation of the neural cortices involved in EF.

In conclusion, this study aimed to investigate the efficacy of rTMS stimulation on site-specific targets to improve EFs in patients with MCI.

## 2. Materials and Methods

### 2.1. Patient Recruitment

Eleven patients diagnosed with MCI according to Petersen’s criteria [38] (male/female: 7/4; mean age: 75 ± 3.71 years (69–80); years of education: mean = 12.09 ± 4.287 [8,9,10,11,12,13,14,15,16,17,18,19,20,21,22,23]) were enrolled in this study from the Neuropsychological and Speech Therapy Unit of the Neurocenter of the Southern Switzerland (EOC), Lugano, Switzerland.

All recruited patients presented a Mini Mental State Examination (MMSE) score ≥ 24/30 [39,40] and a Token test ≥ 26.5 to ensure they had the ability to understand the study procedures [40]. Moreover, they must have had an age between 50 and 85 years old at the time of informed consent; had at least 5 years of education or work experience to exclude mental deficits other than MCI; met Petersen’s criteria for mild cognitive impairment; and had a clinical dementia rating global score of 0.5 and a score < 29 for the Beck depression inventory to exclude major depression that could compromise a patient’s ability to engage in the study. Patients were excluded from the study if they presented at least one of the following main exclusion criteria: Clinically significant unstable psychiatric illness requiring treatment with neuroleptic; transient ischemic attack, stroke, or any unexplained loss of consciousness or severe ongoing stressor within 1 year prior to screening; history of seizure within 10 years prior to screening; recent history of alcohol or substance abuse or use of cannabinoids; or severe head trauma in the past.

In addition, contraindications to having TMS treatment were investigated using a standard safety questionnaire (TMS safety questionnaire, edited by Rossi et al. 2009 [41], aimed at screening potential subjects’ risk of adverse events during TMS treatment).

Nineteen MCI patients were screened; 11 of them satisfied criteria of eligibility and were included in the study.

**Figure 1:**
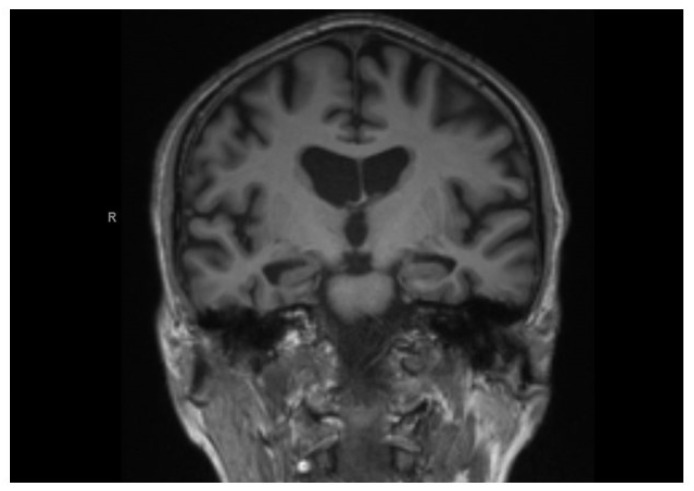
Magnetic resonance imaging picture in a patient enrolled in the study with abnormal MTA score.

All patients underwent cerebral magnetic resonance imaging that was viewed by an expert neuroradiologist who described the Fazekas and MTA scores, and patients affected by tumor, a focal lesion, or an infection/inflammatory disease were excluded (an example of an MRI sequence is shown in Figure 1).

To better define their cognitive profile, patients with MCI completed a neuropsychological battery for mild cognitive deficits [42]. Nine patients had multiple-domain MCI, and two of them had single-domain amnestic MCI. Furthermore, seven patients met the criteria for MCI due to AD [1]: four were positive for all cerebrospinal markers (i.e., A-beta 1–42, Tau total, p-Tau), one for amyloid on PET, and two had selective atrophy of the medial temporal lobe (Table 1).

The text continues here

The local ethics committee approved the study, and all participants provided written informed consent before their involvement in any study procedure.

### 2.2. Intervention

**Figure 2:**
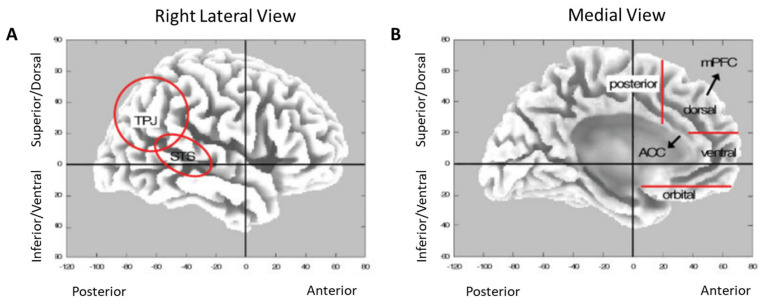
(**A**,**B**) The anatomy of the human brain and the major areas involved in social cognition, placed in x–y–z stereotactic atlas, from F. V. Overwalle, Human Brain Mapping 30:829–858 (2009).

All patients received 2 weeks of excitatory rTMS stimulation of the right temporo-parietal junction (RTPJ) and medial prefrontal cortex (MPFC) (see Figure 2 for further details). Each week of rTMS treatment consisted of five sessions (30 min each, once per day; in each session, 15 min were dedicated to the RTPJ stimulation and 15 min to the MPFC stimulation). rTMS was delivered through a 70 mm cooled coil connected to a Magstim Rapid 2 stimulator (Magstim Co., Whitland, UK.).

Before starting rTMS treatment, the resting motor threshold (rMT) was established for each subject with an electromyography (EMG) exam (mean = 55.45%; SD = 4.6). The stimulation intensity used during the experiment was set to 100% of the rMT of each subject. Trains of rhythmic high-frequency (20 Hz) rTMS were delivered in short periods (3 s duration) separated by longer periods (28 s) of no stimulation for each daily session. The total number of pulses per session was 2000. These parameters were consistent with the safety recommendations for rTMS [41].

**Figure 3:**
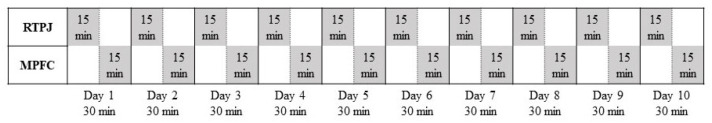
Schematic diagram of the location of stimulation. Abbreviations: RTPJ = right temporo-parietal junction; MPFC = medial prefrontal cortex.

Skull landmarks (nasion, inion, and two preauricular points) and 33 points providing a uniform representation of the scalp were identified with a stylus pen. Thus, the target points expressed in Talairach space were inserted manually. Coordinates in Talairach space [43] were automatically estimated by the SoftTaxic Navigator from an MRI-constructed stereotaxic template. The size of the stimulation was equal for the two target regions (median: 54%, range 52–65%). During the rTMS treatment, the coil was positioned over the RTPJ and MPFC and constantly monitored using the SoftTaxic neuronavigation system (EMS, Bologna, Italy) coupled with a Polaris Vicra infrared camera (NDI, Waterloo, Canada). We first stimulated the RTPJ and sequentially the MPFC. Figure 3 shows a schematic diagram of the stimulation location.

### 2.3. Evaluation

Before the TMS treatment, participants underwent a structured interview with a neuropsychologist to investigate sociodemographic and clinical variables. Cognitive and emotional assessments were performed twice: at baseline (T0), before the start of treatment, and after 10 rTMS sessions (T1), in order to evaluate the immediate impact of the stimulation on cognitive functioning [25].

The battery was composed as follows:

Cognitive status assessment: A battery of validated tests was used to explore the patients’ cognitive functioning. The Montreal cognitive assessment (MoCA) is a widely used screening instrument, accepted as a cognitive assessment tool for MCI patients, and generates a total score ranging from 0 (worst performance) to 30 (best performance). It includes subtasks to assess six cognitive domains: memory (assessed with delayed recall of five nouns; score range: 0–5); visuospatial abilities (assessed by clock-drawing and cube-copy tasks; score range: 0–4); executive functions (assessed by a brief version of the TMT-B, a phonemic verbal fluency, and a two-item verbal abstraction task; score range: 0–4); attention, concentration, and verbal memory (assessed by target detection, subtraction, and forward and backward span tasks; score range: 0–6); language (assessed by naming, repetition, and phonemic fluency tasks; score range: 0–6); temporal and spatial orientation (assessed with specific queries, score range [1]: 0–6 [43].

Executive functions were investigated using the trial making test (TMT) (A and B) to measure processing speed, sequencing, attention, mental flexibility, and psychomotor speed. In TMT A, the subject is asked to connect 25 circled numbers in the correct ascending order, whereas in TMT B, the subject is invited to alternately connect circled numbers (in ascending order) and circled letters (in alphabetical order). As the primary outcome variable was the time the subject took to complete the tasks, the participant needed to connect the items as quickly as possible [44].

A frontal assessment battery (FAB) was also administered. This battery assesses global executive dysfunction, generating a total score ranging from 0 (worst performance) to 18 (best performance). It comprises six subtasks assessing conceptualization, mental flexibility, motor programming, sensitivity to interference, inhibitory control, and environmental autonomy [45].

Emotional assessment: Depressive symptoms were evaluated using the geriatric depression scale (GDS), a widely used validated scale composed of 30 dichotomous items (yes/no), with scores ranging from 0 (no depression) to 30 (severe depression) [46].

Apart from the MoCA subtasks, all scores were corrected for age and education.

### 2.4. Statistical Analysis

Variables are reported as mean and standard deviation, median and interquartile range, or count and relative frequencies. Nonparametric tests were used to compute differences across the two time points. Comparisons between “before TMS treatment” (T0) and “after TMS treatment” (T1) across the cognitive and emotional variables were performed using the Wilcoxon signed-rank test. Due to the exploratory and descriptive nature of the study, no correction for multiple comparisons was performed.

All statistical analyses were performed using the IBM SPSS statistical software for Windows (version 23.0; IBM Corp., Armonk, NY, USA). Statistical significance was set at *p* < 0.05.

## 3. Results

Apart from one patient who did not complete the TMT-B test at T0 and T1, all participants completed the set of assessment.

**Figure 4:**
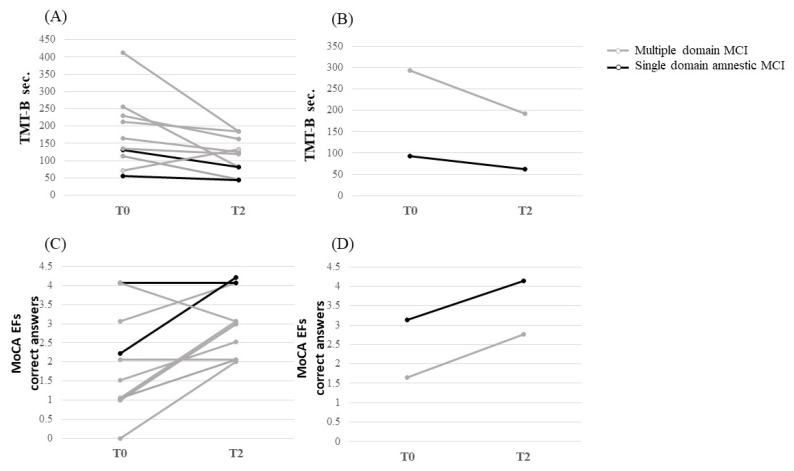
Ten days of transcranial magnetic stimulation effect on executive functions in multiple domain and single domain, amnestic MCI. (**A**) Executive functions assessed with the trial making test—part B (TMT-B) by patient; (**B**) executive functions assessed with the TMT-B by patients with multiple domain and single domain, amnestic MCI; (**C**) executive functions assessed with the Montreal cognitive assessment (MoCA) subtasks by patient; (**D**) executive functions assessed with MoCA subtasks by patients with multiple domain and single domain, amnestic MCI. Abbreviations: TMT-B = trial making test—part B; MoCA EFs = Montreal cognitive assessment executive function subtasks.

At baseline, six multiple-domain MCI patients’ (54.5%) were compromised (MCI_EF CI) in at least one of the two tests used to investigate attention-executive function (TMT and FAB). MCI patients showed a significant improvement in the MoCA executive function subtask (T0 vs. T1, *p* = 0.015) and TMT-B (T0 vs. T1, *p* = 0.028) after treatment; both amnestic and multiple-domain MCI patients showed an improvement in EFs (Figure 4). Specifically, 8 patients improved in the MoCA executive function subtask, while 10 did in the TMT-B, independently from the type of impairment. Conversely, no significant improvement was found in the MoCA memory subtask (T0 vs. T1, *p* = 0.414). Interestingly, we found a tendency to improve global cognitive function, as measured by the MoCA (*p* = 0.049) (Table 2). No corrections for multiple comparisons were calculated.

After ten days of TMS treatment, 5/6 (83%) MCI EF CI patients showed an improvement in their attention-executive functions.

## 4. Discussion

In this study, we found that high-frequency rTMS intervention on the RTPJ and MPFC improved attentional-executive functioning in patients with MCI.

The study showed a significant effect of RTPJ and MPFC stimulation on the EFs. In detail, there is an improvement in the EF part of the MoCA and TMT B (see Table 2), while nonsignificant change was found in the FAB. This is probably due to the composite nature of the battery, which includes the evaluation of six subdomains, and possibly hides specific improvements; this null result might be amplified by the limited number of study participants.

A nonsignificant improvement was found in the memory subtask of the MoCA and TMT A. Furthermore, the EF enhancement would not seem to be attributable to the well-known “exercise or training effect” phenomenon. In fact, parallel versions of the MoCA test were used to avoid the test-retest issue, and evidence from Basso et al. [47] shows no improvement in TMT performance across a period of 12 months. This is likely related to the fact that the strategies required for successful TMT A and B performance are relatively simple and may require acquired cognitive skills.

The improved score in TMT B demonstrates the relevance of our rTMS therapy on mental flexibility and switching capacity, which is known to be related to the dorsolateral and medial frontal regions of the brain [48]. Our study was conducted in an elderly and well-characterized population affected by MCI (Table 1). This population usually has few therapeutic opportunities [21] for treating EF deficits.

The RTPJ plays a crucial role in multimodal sensory integration [49,50] and reorienting attention [51], while the MPFC is strongly implicated in executive functions [52,53] and working memory [53]. In addition, both have a dynamic role in the DMN [54] and VAN [34]. Moreover, the RTPJ and MPFC are part of the mentalizing system, which complements the mirror system for social understanding, that is, inferring others’ intentions [55]. The substantial difference between the two is that the mentalizing system is mainly used when the intentions of others cannot be understood by simple visual cues and must therefore be intuited by reasoning about the interlocutor’s possible thoughts and beliefs [56]. Thus, the RTPJ and MPFC are particularly relevant in attention-executive skills, which are indispensable for social cognition. Consequently, these two specific cognitive functions share, at least in part, the same neuronal networks. Indeed, regarding our research population, recent studies have highlighted the significant impact of cognitive functioning and EF on social cognition performance in AD patients [57,58].

A large body of evidence showed the role of rTMS in the modulation of excitability and plasticity in a targeted cortical region [59] and demonstrated its broader effects across the networks connected to that region [60,61,62]. Moreover, the regulation of dysfunction within and between functional networks is assumed to be the potential mechanism of action of the therapeutic effects of rTMS [63,64]. Indeed, rTMS modulates brain plasticity and might trigger long-term effects in both the stimulated area and the related networks.

Based on this evidence, we can hypothesize that our preliminary clinical findings are the expression of neuromodulation of pathways underpinning attention-executive skills to redirect attention toward behaviorally relevant stimuli.

Future studies of MRI connectivity are warranted to clarify the modification of cerebral mechanisms involved in the positive response to TMS stimulation on RTPJ and MPFC.

To the best of our knowledge, this is the first time RTPJ and MPFC have been stimulated in patients with MCI to verify their impact on EF. Some studies have successfully tested the effectiveness of transcranial magnetic stimulation [19,65] in areas traditionally associated with EF, such as the dorsolateral prefrontal cortex. These areas have circuits that connect the hippocampus (memory) and mood-related aspects [66]. From the previous literature, we know that awareness, social withdrawal, and apathy [67] are more dependent on the circuits we stimulate, even if those aspects have not been directly investigated. It remains interesting to understand whether cognitive elements play a causal role or represent an overlapping disorder [58,68].

The most important limitations of these preliminary findings are the small size of the study group and the absence of a placebo group. However, the highlighted ameliorative effect is not cross-cutting across all cognitive domains but is specific to EF. Another limitation is the restricted number of tests administered to evaluate EF. Although many studies have verified the efficacy of treatments aimed at improving EF [69,70], TMT could have been combined with the Wisconsin Card Sorting Test (WCST) [71] or the category test, which are considered excellent tests for analyzing this specific cognitive function, even if they are less effective in a therapeutic trial.

The strength of our study is the selection of patients. Participants were enrolled after an accurate medical history; a specific neuropsychological battery for MCI [42]; an MRI read with visual scales for Fazekas and medial temporal atrophy [72]; and, in half of the patients, a cerebrospinal fluid examination with positive markers of both neuronal injury and A-beta deposition [1].

This study allows future researchers to replicate our data and test the simultaneous effect of rTMS therapy on social cognition.

Our preliminary results also open new hypotheses for studying DMN and VAN networks in patients with MCI. Additionally, it could promote further research in the early stages of the disease, when nonthreatening but effective therapies are needed.

These results need to be confirmed in larger, placebo-controlled trials, before further conclusions on the value of this therapeutic approach can be drawn.

## Figures and Tables

**Table 1:** Participants socio-demographical, neuropsychological, and cerebrospinal fluid and MRI biomarkers characteristics.

Subject	Age	Sex (0 = Female; 1 = Male)	Education (Years)	MCI Type	MoCA Score	Liquor Abeta 1–42	Liquor Tau total	Liquor p-tau	Fazekas/MTA
**1**	79	1	8	Multiple-domain	17 *				1/2 *
**2**	74	1	8	Multiple-domain	16 *	/	/	/	0/2 *
**3**	77	1	13	Multiple-domain	22 *	555 *	496 *	94 *	0/2 *
**4**	76	1	8	Multiple-domain	15 *	/	/	/	1/1
**5**	76	1	9	Multiple-domain	18 *	/	/	/	3 */1.5
**6**	69	0	12	Multiple-domain	22 *	511 *	824 *	191 *	1/0
**7**	70	1	13	Single-domain amnestic	25 *	/	/	/	1/1
**8**	80	0	13	Single-domain amnestic	21 *	/	/	/	2 */1
**9**	71	0	23	Multiple-domain	17 *	609 *	590 *	103 *	0/1
**10**	78	0	13	Multiple-domain	20 *	584 *	517 *	100 *	1/1
**11**	77	1	13	Multiple-domain	20 *	/	/	/	/

Abbreviations: MRI, magnetic resonance imaging; MCI, mild cognitive impairment; MOCA, Montreal Cognitive Assessment./: The symbol “/” indicates nonpathological values. * When pathological.

**Table 2:** Comparison between T0 and T1 for cognitive and emotional variables.

	T0 (Prior rTMS Treatment) Median (IQR)	T1 (after TMS Stimulation) Median (IQR)	*p*
MoCA—Global score	21.0 (18.5–23.0)	23.0 (21.0–25.0)	0.049
MoCA—Memory	0.0 (0.0–0.5)	0.0 (0.0–1.25)	0.414
MoCA—Visuospatial abilities	3.0 (2.0–3.5)	3.5 (2.8–4.0)	0.102
MoCA—Executive functions	2.0 (1.0–3.0)	3.0 (2.0–4.0)	0.015
MoCA—Attention	6.0 (5.0–6.0)	6.0 (5.0–6.0)	0.414
MoCA—Language	4.0 (4.0–6.0)	5.0 (3.8–5.3)	0.340
MoCA—Orientation	5.0 (3.0–6.0)	5.0 (3.8–6.0)	0.792
TMT-A	41.0 (23.0–66.5)	27.0 (19.8–48.8)	0.398
TMT-B	134.0 (92.0–243.5)	121.0 (73.0–168.3)	0.028
TMT B-A	105.0 (44.0–180.0)	73.5 (28.0–102.3)	0.066
FAB	16.0 (13.5–17.0)	15.5 (13.8–16.3)	0.952
GDS	6.0 (4.5–11.5)	6.5 (4.0–8.5)	0.641

Abbreviations: MOCA, Montreal cognitive assessment; TMT, trial making test; FAB, frontal assessment battery; GDS, geriatric depression scale.

## Data Availability

The raw data supporting the conclusions of this article will be made available by the authors without undue reservation.
